# Effects of Early Nutrition on Premature Infants

**DOI:** 10.3390/nu17101648

**Published:** 2025-05-12

**Authors:** Rita C. Silveira, Renato S. Procianoy

**Affiliations:** Hospital de Clínicas de Porto Alegre, Universidade Federal do Rio Grande do Sul, Porto Alegre 9045007, Brazil; drarita.c.s@gmail.com

Early nutrition plays a crucial role in both the short- and long-term health outcomes of premature infants, particularly those born with very low birth weight or extremely low gestational age. These infants are especially vulnerable due to their immature organs, limited nutrient reserves, and high metabolic demands [1,2].

A recent cohort study assessed the association between neonatal protein intake and brain structure at 7 years of age in children born very preterm, comparing two groups before and after a change in Neonatal Intensive Care Unit (NICU) nutritional protocol that increased protein intake. Although the group exposed to the higher-protein protocol showed reduced relative brain volume and cortical thinning in the occipital and parietal regions, absolute brain volumes were comparable between the groups. Higher neonatal intake of protein, fat, energy, and breast milk was associated with a more mature white matter microstructure, as indicated by higher fractional anisotropy and lower diffusivity on diffusion tensor imaging. These findings suggest that increased early protein intake may promote white matter maturation, with effects persisting into childhood [2].

In terms of nutritional recommendations for preterm neonates, human milk is the preferred form of enteral nutrition. The hierarchy of options includes the following: (1) raw mother’s milk, (2) pasteurized mother’s milk, and (3) pasteurized donor milk. Breastfeeding enhances mother–infant bonding and positively impacts neurodevelopment, highlighting the importance of family-centered care and breastfeeding support in NICUs [3].

Despite digestive immaturity, the early initiation of enteral feeding with human milk is strongly recommended—even for extremely preterm infants (<28 weeks’ gestation or <1000 g birth weight). Delayed feeding, slow advancement, and routine gastric residual volume checks are outdated practices that prolong the need for parenteral nutrition and increase the risk of complications [4].

The fortification of human milk is essential for very low birth weight infants, as standard volumes of human milk alone may not meet their high nutritional requirements. Early fortification has been linked to improved growth trajectories without increasing the risk of metabolic disorders later in life. Studies show that higher protein fortification (≥1.4 g/100 mL) enhances growth without documented adverse effects. Individualized fortification—guided by an analysis of the mother’s own milk (MOM) or pasteurized donor human milk (PDHM)—is a promising strategy to optimize nutrient intake in preterm infants. Although still emerging, evidence suggests benefits for clinical outcomes, particularly in cases of growth faltering.

Before conducting milk analysis, it is essential to rule out inadvertent fat losses during handling (e.g., milk transfers or prolonged pump feeding) and to ensure standard fortification has been fully optimized per local protocols. Milk analysis may then support targeted adjustments or prompt investigation of other causes of inadequate growth, such as increased metabolic demands, malabsorption, or genetic disorders. Clinicians should exercise caution when discussing nutrient analysis results with families to avoid undermining confidence in the adequacy of MOM. Accurate interpretation requires the use of true protein values (excluding non-protein nitrogen), metabolizable energy estimates, and representative samples. Currently, the lack of standardized methods for milk nutrient measurement and labeling limits comparability across settings, highlighting the need for further rigorous research [5,6].

Feeding strategies should include early colostrum expression (ideally within the first 6 h) and small, frequent doses administered via oral syringe. Standardized enteral feeding protocols help improve nutritional outcomes. Feed advancement should be based on birth weight and increased as tolerated. For extremely preterm infants, early fortification (starting on day 2) can improve linear growth and head circumference without increasing fat mass. Early feeding with human milk supports gastrointestinal development, helps establish a beneficial gut microbiome, and reduces the risk of necrotizing enterocolitis (NEC), a severe and potentially fatal intestinal condition [1,3]. Colostrum, rich in immunological components, plays a crucial role in early gut protection and immune system priming. Delayed or overly cautious advancement of feeds may result in nutritional deficits, poor growth, and suboptimal developmental outcomes.

Bolus feeding is generally preferred over continuous infusion, except in cases of intestinal dysmotility. Routine gastric residual checks should be avoided in favor of clinical assessment. High-volume feeding (>180 mL/kg/day) supports better growth and neurodevelopment, though special consideration is needed in conditions such as bronchopulmonary dysplasia or patent ductus arteriosus. Nutritional decisions should be guided by anthropometric monitoring based on gestational age and clinical comorbidities [1,3,4].

Early enteral nutrition—preferably with human milk—plays a pivotal role in reducing the need for parenteral nutrition (PN) in preterm infants, thereby minimizing associated risks. Early enteral feeding promotes gastrointestinal maturation, hormonal and metabolic adaptation, and supports neurodevelopment. The prompt initiation of human milk-based enteral nutrition can decrease the duration and volume of PN required, facilitating earlier removal of central lines and reducing the risk of central line-associated bloodstream infections, liver dysfunction, and sepsis [1,3,5].

Additionally, the gradual advancement of enteral feeds helps prevent refeeding syndrome—a metabolic disorder marked by electrolyte imbalances, particularly hypophosphatemia, that can occur when nutrition is reintroduced after undernutrition. Preterm infants, especially those ≤32 weeks’ gestation, <1500 g, or with severe intrauterine growth restriction, are at elevated risk of refeeding syndrome during early PN due to limited nutrient stores and high metabolic demands. Thus, a balanced approach combining early enteral and PN—including the timely provision of amino acids and lipids and adequate electrolyte supplementation—is essential to ensure safe nutritional progression, metabolic stability, and optimal neurodevelopmental outcomes [1,2,4].

In conclusion, early nutrition in premature infants is more than a feeding strategy—it is a critical form of early intervention. Timely, appropriate, and individualized nutritional care can profoundly improve survival, growth, and developmental trajectories. The focus should be on early human milk-based enteral nutrition, with fortification when needed and minimal dependence on PN. Practices such as early colostrum expression and skin-to-skin care may improve breastfeeding rates and contribute to sustained developmental benefits and stronger mother–infant bonding.

## Data Availability

Not applicable.
